# Aerosolized Dornase Alfa (DNase I) for the Treatment of Severe Respiratory Failure in COVID-19: A Randomized Controlled Trial

**DOI:** 10.1093/ofid/ofaf246

**Published:** 2025-04-24

**Authors:** Per Åkesson, Lisa Mellhammar, Magnus Rasmussen, Malin Inghammar, Sara Jesperson, Fredrik Månsson, Elin Economou Lundeberg, John Walles, Martin Wallberg, Attila Frigyesi, Adam Linder

**Affiliations:** Division of Infection Medicine, Department of Clinical Sciences Lund, Lund University, Lund, Sweden; Department of Infectious Diseases, University Hospital, Lund, Sweden; Division of Infection Medicine, Department of Clinical Sciences Lund, Lund University, Lund, Sweden; Department of Infectious Diseases, University Hospital, Lund, Sweden; Division of Infection Medicine, Department of Clinical Sciences Lund, Lund University, Lund, Sweden; Department of Infectious Diseases, University Hospital, Lund, Sweden; Division of Infection Medicine, Department of Clinical Sciences Lund, Lund University, Lund, Sweden; Department of Infectious Diseases, University Hospital, Lund, Sweden; Clinical Studies Sweden–Forum South, Skåne University Hospital, Lund, Sweden; Department of Infectious Diseases, Skåne University Hospital, Malmö, Sweden; Department of Infectious Diseases, Central Hospital of Kristianstad, Kristianstad, Sweden; Department of Infectious Diseases, Central Hospital of Kristianstad, Kristianstad, Sweden; Clinical Infection Medicine, Department of Translational Medicine, Lund University, Malmö, Sweden; Department of Pulmonary Diseases, Skåne University Hospital, Lund, Sweden; Department of Anesthesiology and Intensive Care, Skåne University Hospital, Lund, Sweden; Division of Infection Medicine, Department of Clinical Sciences Lund, Lund University, Lund, Sweden

**Keywords:** aerosolized DNase I, COVID-19, respiratory failure, sepsis, NETs

## Abstract

**Background:**

Lung injury in COVID-19 is characterized by neutrophil invasion and the release of neutrophil extracellular traps (NETs). An aberrant NET formation may induce local inflammation and increase sputum viscosity. Inhalation of DNase I (dornase alfa) is a treatment option that degrades NETs in the airways. Previous case series have indicated positive clinical effects of inhaled dornase alfa.

**Methods:**

Patients admitted to the hospital with acute COVID-19 and hypoxia (oxygen saturation <90%) were randomly assigned to receive aerosolized dornase alfa twice daily for 5 days or a placebo in addition to standard of care. The primary outcome was discharge from the hospital or an oxygen saturation >93% without respiratory support.

**Results:**

In total, 76 patients were randomized. The study was stopped when the Omicron virus variant appeared. The clinical response rate did not differ between patients receiving the active substance and placebo. Secondary outcomes were similar across groups, such as mortality, a new episode of hypoxia, length of stay in the hospital, and adverse events. A subanalysis of patients older or younger than 65 years showed no differences in primary or secondary outcomes.

**Conclusions:**

Aerosolized dornase alfa failed to improve hypoxia in hospitalized patients with acute COVID-19. The study was conducted during a time of heterogeneity in viral variants and vaccination status of participants. Whether dornase alfa affects the outcomes in other respiratory infections requires further study.

During the first 2 years of the COVID-19 pandemic, severe respiratory dysfunction was common among hospital-bound patients. The leading symptom was hypoxia, which in some cases worsened to acute respiratory distress syndrome (ARDS). Most patients admitted to the hospital required oxygen support, and some developed critical disease, including ARDS [1]. The progression to ARDS is characterized by hypercoagulability and reduced pulmonary perfusion [2]. In addition, inflammatory edema is largely mediated by proinflammatory cytokines released by macrophages and granulocytes.

Another proposed mechanism coupled to neutrophil invasion in COVID-19 is the formation of NETs (neutrophil extracellular traps). These are extrusions of neutrophil DNA and attached granule proteins that may enable trapping and killing of microbes. However, they are cytotoxic to lung endothelial and epithelial cells and can induce clot formation and vascular occlusion in the lungs [3, 4]. This suggests a NET-driven hindrance of gaseous exchange during infection. In clinical studies, higher NET levels in the blood and respiratory tract have been found in patients with infection-related ARDS [5–7].

NETs can be dissolved with DNase I, and preclinical studies have suggested that the removal of NETs with DNase I is beneficial in bacterial and viral infections [8, 9]. In severe COVID-19, DNase I could target dysregulated NET formation and affect the prothrombotic state and the formation of highly viscous sputum. Dornase alfa (Pulmozyme; Roche) is the recombinant form of human DNase I. It is currently used in humans to improve sputum clearance in patients with cystic fibrosis. Early in 2020, dornase alfa was used off-label in a pilot study of 5 patients with severe hypoxia who were hospitalized with COVID-19. Treatment was administrated via nebulizer, resulting in clinical improvement and less need for supplemental oxygen [10]. Immunofluorescence and proteome analyses showed that initial elevated levels of NETs in sputum were reduced within 3 days of treatment. This promising result was the basis for the present randomized controlled trial designed to investigate the efficacy of aerosolized dornase alfa on time to cessation of oxygen therapy in patients with severe COVID-19.

## METHODS

### Study Design and Participants

This investigator-initiated single-blinded trial based on a randomized placebo-controlled design was conducted in Sweden between June 2020 and January 2022. Patients were enrolled at Skåne University Hospital and the Central Hospital of Kristianstad in southern Sweden. Ethics approval for the trial was obtained from the Swedish ethics review authority (reference 2020-02218). The trial was registered at ClinicalTrials.gov (NTC04541979) before patient enrollment. All procedures followed the ethical standards of the 1964 Helsinki Declaration and its later amendments. Written informed consent was obtained from all participants before any study-related procedures.

Adults (≥18 years of age) were eligible for inclusion if they were admitted to a hospital ward or intensive care unit (ICU), were diagnosed with COVID-19 by a positive result from a polymerase chain reaction test, and had oxygen saturation ≤90% without supplemental oxygen. The exclusion criteria included chronic obstructive pulmonary disease stage III or IV or comparable chronic respiratory disease and a known or suspected allergy against dornase alfa.

### Randomization and Masking

Study participants were randomized consecutively as they were found eligible for inclusion by the clinicians caring for the patients. A centralized, internet-based, computer-generated allocation tool was used. Allocation was performed in a 1:1 ratio to either the study drug or the placebo. Randomization was stratified by age (<65 or ≥65 years) to account for the known worse prognosis in older groups and performed with blocks of varying sizes. The clinical team caring for the patient was not blinded to the assigned intervention. Study participants, their relatives, trial statisticians, and outcome assessors were blinded to the assigned intervention.

### Intervention

Participants allocated to receive the active substance were given 2.5 mg (2.5 mL) of dornase alfa (Pulmozyme) as an inhaled medication by a nebulizer twice daily. Treatment was administered for 5 days, which equals 10 doses and a total dose of 25 mg of dornase alfa per participant. In the placebo group, 2.5 mL of isotonic saline (NaCl 0.9%) was administered identically twice daily for 5 days. The administration of the study drug and placebo was done by nurses at the study center within the hospital routine.

### Outcomes

The primary outcome was time to cessation of oxygen therapy or discharge from hospital. The level of hypoxia was measured twice daily by pulse oximetry after supplemental oxygen given by nasal cannula or mask was stopped. When oxygen saturation was stable at a level >93% for 24 hours, as measured at least every 6 hours, the primary outcome was considered to have been reached. In addition, the primary outcome was met if participants were discharged from the hospital during the 24-hour measurement period after cessation of oxygen treatment. Participants needing a ventilator, high-flow nasal cannula (HFNC) oxygen, or supplemental oxygen with a flow >5 L/min were considered to have a saturation ≤93%.

The secondary outcomes included 28-day mortality, days on a mechanical ventilator, days undergoing HFNC oxygen treatment, length of stay in the hospital, length of stay in the ICU, a new episode with saturation ≤93% (after the primary end point had been met), and total number of days without need for supplemental oxygen. Adverse events (AEs) were also registered as secondary outcomes. These were captured at the daily physical examination and by reviewing patient charts.

Primary and secondary outcome measures were evaluated within the 28-day study period, with an extension of up to 360 days for evaluating long-term mortality.

### Statistical Analysis

A size calculation was conducted to achieve 80% power to detect differences in the active arm vs the control group at a 5% significance level. Based on the primary end point, time to cessation of oxygen therapy or discharge from hospital, a total sample size of 98 participants would provide sufficient power if the number of days was 15 for placebo and 8 for inhaled dornase alfa. One statistician performed all analyses independently according to the statistical analysis plan before unblinding the data. The analyses were performed according to an intention-to-treat principle. Continuous variables are presented as mean (SD) if the distribution is symmetric and median (IQR) otherwise. Count data are presented as median (IQR), while categorical data are presented as number (percentage). All statistical tests are 2-sided. The primary end point was analyzed by the log-rank test with a significance level of 5% and presented as a Kaplan-Meier plot. The hazard ratio and 95% CI were reported from the Cox regression analysis. Patients who did not yet achieve clinical improvement during the 28-day study period or died during the study were censored at 28 days. The secondary end points regarding the proportion of deaths and the proportion of participants with a new episode with saturation ≤93% (after the primary end point had been met) were reported separately in each group and as the absolute difference between the arms with 95% CI. Secondary end points, measured in days, are reported separately for each arm, and the median difference between the arms with 95% CI was calculated by the Hodges-Lehman procedure. These results are reported for all patients (intention to treat) and separately for survivors.

In addition, all analysis was performed in subgroups specified by age (<65 and ≥65 years).

## RESULTS

### Patients’ Characteristics

**Figure 1.**
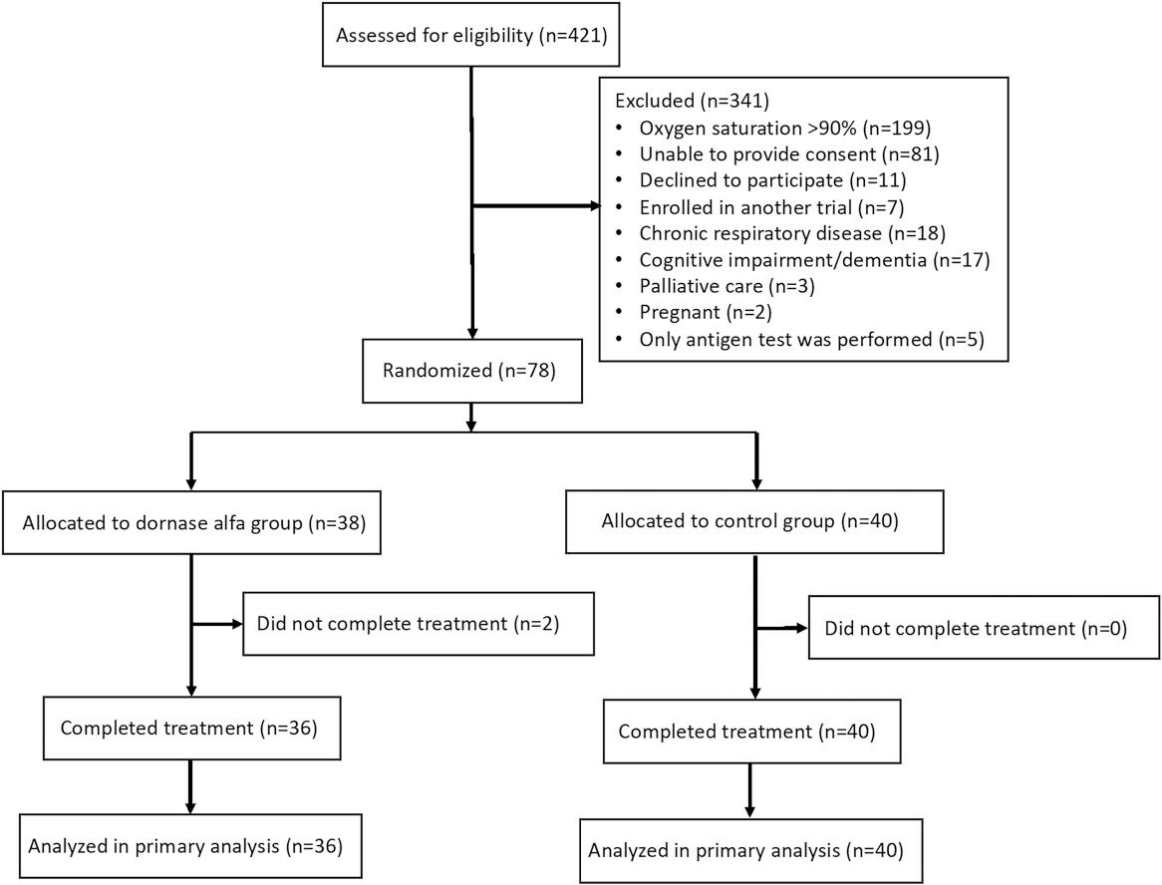
Participant enrollment, randomization, and follow-up.

Between 4 June 2020 and 11 January 2022, eligibility was assessed in 421 adult patients admitted with COVID-19 in 2 Swedish hospitals. The last follow-up for the last patient was on 6 January 2023. An overall 202 patients did not meet the inclusion criteria. The most frequent reason was oxygen saturation >90%. In total, 128 patients fulfilled 1 or more exclusion criteria, and 11 declined to participate (Figure 1). Seventy-six patients were randomized for this study: 36 were randomly assigned to receive aerosolized dornase alfa, and 40 were assigned to the control group. All patients completed the trial treatment. The clinical and demographic characteristics were well balanced between the treatment groups at baseline (Table 1).

**Table 1.** Baseline Characteristics

Variable	Dornase Alfa (n = 36)	Placebo (n = 40)
Age, y, mean (SD)	60.1 (13.7)	60.8 (11.8)
Male	23 (63.9)	26 (65.0)
Ethnicity		
Caucasian	33 (91.7)	34 (85.0)
Other ethnicity	3 (8.3)	5 (15.0)
Body mass index, kg/m^2^, mean (SD)	30.00 (5.0)	31.14 (5.7)
Comorbidity		
COPD stage I or II	2 (5.6)	2 (5.1)
Hypertension	14 (38.9)	19 (47.5)
Diabetes	5 (13.9)	6 (15.0)
Cardiovascular disease	5 (13.9)	8 (20.0)
Renal disease	2 (5.6)	1 (2.5)
Respiratory disease	4 (11.1)	6 (15.0)
Liver disease	0 (0.0)	0 (0.0)
Malignancy	2 (5.6)	1 (2.5)
Immunodeficiency	0 (0.0)	3 (7.5)
Connective tissue disease	1 (2.8)	2 (5.0)
Concurrent medication		
Corticosteroids	31 (86.1)	38 (95.0)
Anticoagulants	36 (100.0)	39 (97.5)
Antibiotics	23 (63.9)	27 (67.5)
Other antiviral treatments	5 (13.9)	7 (17.5)
Antihypertensive	13 (36.1)	20 (50.0)
Antidiabetics	8 (22.2)	7 (17.5)
Statins	9 (25.0)	10 (25.0)
Immunosuppressants	2 (5.6)	2 (5.0)
Respiratory support during study period		
Any type of support	21 (58.3)	25 (62.5)
Mechanical ventilator	6 (16.7)	6 (15.0)
Noninvasive ventilation	3 (8.3)	4 (10.0)
High-flow nasal cannula	21 (58.3)	25 (62.5)

Data are provided as No. (%) unless noted otherwise.

Abbreviation: COPD, chronic obstructive pulmonary disease.

The mean age of the patients was 60.5 years; 36.8% were ≥65 years old. Most patients were given anti-inflammatory and anticoagulant treatments according to the national guidelines for severe COVID-19. In addition, 90.8% received corticosteroids and 98.7% anticoagulants. All participants were randomized in a regular hospital ward. After randomization, 12 (15.8%) needed mechanical ventilation during the study period and were transferred to the ICU. Excluding patients admitted to the ICU, 34 (44.7%) received supportive oxygen therapy by HFNC or noninvasive ventilation.

### Efficacy

#### Primary Outcome

**Figure 2.**
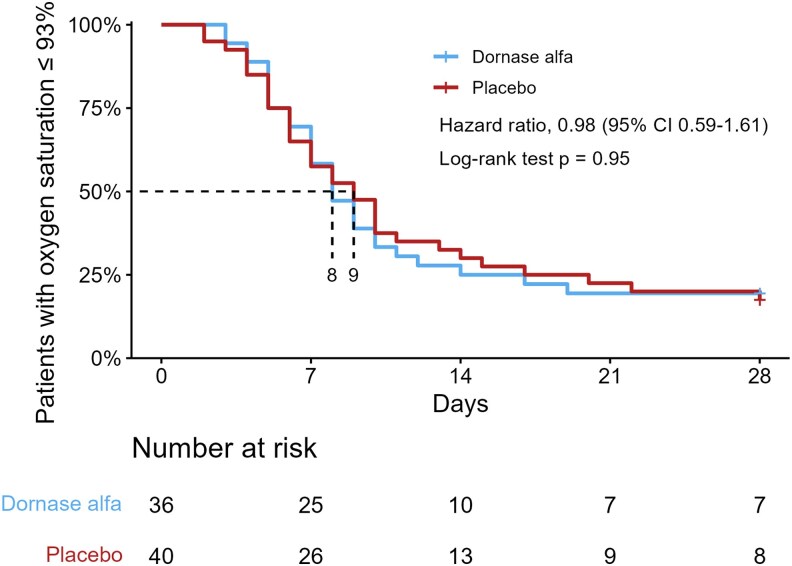
Kaplan-Meier estimates of resolving hypoxia, defined as oxygen saturation >93% without supplemental oxygen for 24 hours. Patients discharged from the hospital were classified as nonhypoxic. The median time in days to resolution of hypoxia is shown under the dashed lines.

Sixty-one patients (80.3%) reached the primary outcome of stable resolved hypoxia (oxygen saturation >93% for 24 hours) or discharge from the hospital. The log-rank test revealed no significant difference between the groups receiving active substance or placebo. This was further confirmed in a Cox regression analysis with a hazard ratio of 0.98 (95% CI, .59–2.1; Figure 2). Analyses of subgroups of patients based on age revealed equivalent results. The hazard ratio was 1.11 (95% CI, .42–2.86) for patients ≥65 years old and 1.06 (95% CI, .59–1.92) for those <65 years old (Supplementary Figure 1).

#### Secondary Outcomes

There were outcome data on 28-day mortality, relapse of hypoxia, length of stay in the hospital and ICU, number of days receiving supportive respiratory treatment, and AEs for all study participants. Results from the analyses of secondary outcomes are presented in Table 2. Eight patients (10.5%) died during the study period, 4 in each treatment group. All deceased patients were ≥65 years old. Six patients (7.9%) deteriorated after reaching the primary outcome with a new episode of hypoxia, defined as oxygen saturation ≤93%. Four of these had been treated with dornase alfa and 2 with placebo. There was no significant difference in length of stay between the groups. Among the 6 patients in each group who needed intensive care, there was no difference in the number of days on a mechanical ventilator or length of stay in the ICU. Forty-six patients (60.5%) were supported by HFNC oxygenation therapy at some stage during the study period. There was no difference in treatment length by HFNC between the 2 study groups.

**Table 2.** Secondary Outcomes

	No. (%) or Median (IQR)		
Secondary Outcome	Dornase Alfa (n = 36)	Placebo (n = 40)	Difference in Proportion (95% CI) or Median (IQR)
Deceased	4 (11.1)	4 (10.0)	1.1 (−12.7, 15.0)
New episode of hypoxia^a^	4 (11.1)	2 (5.0)	6.1 (−6.2, 18.4)
Length of stay, d			
In hospital	7 (5–11)	9 (6–15)	−1 (−4, 1)
Intensive care unit^b^	0 (0–0)	0 (0–0)	0 (0, 0)
Days of respiratory support			
Mechanical ventilator^b^	0 (0–0)	0 (0–0)	0 (0, 0)
High-flow nasal cannula	3 (0–6)	2 (0–6)	0 (−1, 2)
Adverse event			
Blood and lymphatic system disorder	1 (2.8)	0 (0.0)	2.8 (−2.6, 8.1)
Cardiac disorder	4 (11.1)	9 (22.5)	−11.4 (−27.9, 5.1)
Endocrine disorder	2 (5.6)	3 (7.5)	−1.9 (−13.0, 9.1)
Gastrointestinal disorder	5 (13.9)	3 (7.5)	6.4 (−7.5, 20.3)
Hepatobiliary disorder	5 (13.9)	4 (10.0)	3.9 (−10.7, 18.5)
Infection	7 (19.4)	8 (20.0)	−0.6 (−18.5, 17.4)
Nervous system disorder	5 (13.9)	5 (12.5)	1.4 (−13.9, 16.6)
Psychiatric disorder	2 (5.6)	2 (5.0)	0.6 (−9.5, 10.6)
Respiratory disorder	10 (27.8)	11 (27.5)	0.3 (−19.9, 20.4)
Skin and subcutaneous tissue disorder	1 (2.8)	5 (12.5)	−9.7 (−21.3, 1.8)
Vascular disorder	1 (2.8)	3 (7.5)	−4.7 (−14.5, 5.0)

Secondary outcomes were assessed when all patients reached day 28 and are reported separately in each group as the absolute difference (95% CI) between the arms.

^a^New episode of oxygen saturation ≤93% after the primary end point of saturation >93% for at least 24 hours had been met.

^b^Median days for the total study group.

There were 96 AEs from 11 categories: 43 in the dornase alfa group and 53 in the control group. Events were evenly distributed except for cardiac, skin, and vascular disorders, which were more frequent in the control group, and gastrointestinal disorders, which were more prevalent in the intervention group. However, the numbers were small and differences nonsignificant. No serious AEs were reported in the study.

Secondary outcomes were analyzed in the 2 age groups: ≥65 and <65 years. The median length of stay for the older group was longer in the control group at 14 vs 6.5 days, with a median difference of −6 (95% CI, −12 to 0; Supplementary Table 1). In the younger group, a novel episode of hypoxia was registered for 4 patients in the dornase alfa group vs 1 patient in the control group, a difference in proportion of 12.5 points (95% CI, −4.4 to 29.4; Supplementary Table 2). Also, secondary infection as an AE was more prevalent in the treatment group (7 vs 2 patients).

## DISCUSSION

In this prospective, randomized, placebo-controlled trial that enrolled patients with COVID-19 and acute respiratory failure, inhaled dornase alfa did not have any beneficial effects on the resolution of hypoxia. Furthermore, the active substance did not affect mortality, length of hospital or ICU stay, or the need for supportive oxygen therapy.

The basis for the trial was prior studies linking aberrant NET formation to pulmonary disease, thrombosis, and mucus secretions in the airways [11, 12]. Early in the pandemic, a possible role of NETs in COVID-19–induced pneumonia was discussed. In a prospective cohort study of 33 patients with severe COVID-19, levels of NETs were elevated in plasma and correlated to disease severity [13]. A recent multiomics study of more than a thousand patients with COVID-19 showed the importance of NETs in inflammation and suggested that severity was partly driven by NETs [14].

Inhalation of dornase alfa in cystic fibrosis has been an established therapy for a long time. This suggested that it might also be a safe treatment option for patients with COVID-19. The benefit would primarily be to improve ventilation by reducing the DNA-mediated viscosity of sputum. Several small patient studies on inhaled dornase alfa have been published. In 1 case series, 5 patients undergoing invasive ventilation were treated with nebulized dornase alfa. They recovered and were successfully extubated [15]. In a nonrandomized case-control study, 10 patients inhaled dornase alfa for 3 days [16]. Results showed improvement in oxygen saturation after 2 days. However, the effect was not sustained. In a retrospective cohort study of 39 patients who were severely ill with COVID-19, respiratory support requirements were reduced after treatment with aerosolized dornase alfa [17]. In 2022, a nonrandomized Greece study showed lower mortality in patients who received inhaled dornase alfa in combination with immunomodulatory drugs [18]. Control groups were undergoing alternative immunosuppressive treatment and enrolled before the dornase alfa group, making interpretation difficult. The only randomized controlled study preceding the present investigation enrolled 39 patients during 2021 and 2022. Results showed a significant reduction of C-reactive protein in plasma, suggesting an anti-inflammatory effect. Finally, in a pilot study from our center, 5 nonrandomized patients recovered after treatment with nebulized dornase alfa [10]. Moreover, significant proteome changes indicated reduced inflammation in the airways and systemically. Taken together, previous clinical studies on dornase alfa treatment showed hopeful results but were small and of lower quality.

There are some possible explanations for the discouraging results in the present study. First, platelet activation and coagulation factors are interconnected to NET release. Excessive release promotes microvascular dysfunction and thromboinflammation [19]. While successful pilot studies were conducted in the very early phase of the pandemic, the present investigation took place at a time when anticoagulant and corticosteroid treatment had been introduced. These therapies likely reduce NET release and thromboinflammation, thereby diminishing the possible beneficial effects of dornase alfa. Second, the timing of the intervention may not have been optimal. Like immunomodulatory and antiviral treatments for COVID-19, there may be a time window where dornase alfa has a beneficial effect. Finally, the study did not specify the time from disease onset to start of treatment.

The study has several strengths. It was a dual-center randomized controlled trial and entailed a daily follow-up routine for all randomized and admitted patients during the study period. No patient was lost to follow-up. Furthermore, the data on AEs indicate that treatment with dornase alfa is safe. The medication was distributed as part of a normal clinical routine, demonstrating a feasible option for severely ill patients during isolation.

Limitations of the trial include the single-blinded design in which hospital and study personnel were aware of the type of inhalation. Also, the study was terminated before the stipulated number of patients were enrolled. The reason was the introduction of the Omicron virus variant at the beginning of 2022, which led to a scarcity of patients with severe COVID-19. The premature study termination in combination with an expected large difference in outcome between placebo and intervention makes it difficult to exclude a more modest effect of the treatment. This may be especially relevant for the older patient group. Another limitation is the nonconsecutive design and the large number of excluded patients. This may have introduced selection bias. Finally, patients were enrolled for >1.5 years. During this time, viral variants replaced each other, vaccination against COVID-19 started, and different immunomodulatory and antiviral treatments were introduced. This may have affected the result.

## CONCLUSION

In the first randomized controlled trial of inhaled dornase alfa for patients with severe COVID-19, there was no significant effect on respiratory function, mortality, need for supportive oxygen treatment, or length of stay. The study was conducted through a dynamic landscape with shifting viral variants and the introduction of various therapies. Further studies are needed to investigate clinically relevant benefits of dornase alfa in other respiratory infections.

## Supplementary Material

ofaf246_Supplementary_Data

## Supplementary Data

Supplementary materials are available at *Open Forum Infectious Diseases* online. Consisting of data provided by the authors to benefit the reader, the posted materials are not copyedited and are the sole responsibility of the authors, so questions or comments should be addressed to the corresponding author.

## Notes

***Acknowledgments.*** Maria Castilla for outstanding research nurse assistance, Peter Lanbeck for making the study possible, and the Clinic for Infectious Diseases Lund for providing Pulmozyme. Susann Ullén for statistical guidance, Liz Jergle for excellent help with study coordination, and Ulf Malmqvist and Forum Söder for providing swift assistance. Kajsa Videfors and medical students for helping with study patient enrollment and data collection. Louise Qvist, Julia Ehlorsson, and Adam Blomstergren for excellent assistance and patient enrollment.

***Author contributions.*** P. Å. helped initiate the study, enrolled patients, and wrote the manuscript. L. M. helped initiate the study, enrolled patients, and critically revised the manuscript. M. R. wrote the application for ethical permit and critically revised the manuscript. M. I. helped initiate the study, enrolled patients, and critically revised the manuscript. S. J. made the statistical analysis. F. M. included patients in Malmö and critically revised the manuscript. E. E. L. included patients in Kristianstad and critically revised the manuscript. J. W. included patients in Kristianstad and critically revised the manuscript. M. W. enrolled patients at the clinic for respiratory medicine and critically revised the manuscript. A. F. enrolled patients at the ICU and critically revised the manuscript. A. L. initiated and led the study, enrolled patients, analyzed the data, and wrote the manuscript.

***Financial support.*** This work was supported by Swedish government funds for clinical research (VR project grant); Region Skåne clinical research grants (ALF project); and The Swedish Heart and Lung Foundation.
