# Supplementary material for: Aerosolized Dornase Alfa (DNase I) for the Treatment of Severe Respiratory Failure in COVID-19: A Randomized Controlled Trial
Source: Open Forum Infect Dis. 2025 Apr 24;12(5):ofaf246. doi: 10.1093/ofid/ofaf246 (PMC12069806; doi:10.1093/ofid/ofaf246)
Supplement: ofaf246_Supplementary_Data [file ofaf246_supplementary_data.zip › Supplemental Table 1.docx]

Supplemental Table 1: The secondary outcomes for participants aged 65 or over.

| **Secondary outcome** | **Dornase alfa**  **(n=12)** | **Placebo**  **(n=16)** | **Difference in proportion**  **(95% CI) or Median (IQR)** |
| --- | --- | --- | --- |
| Deseased, n (%) | 4 (33.3) | 4 (25.0) | 8.3 (-25.7; 2.4) |
| New episode of hypoxia^a^, n (%) | 0 (0.0) | 1 (6.2) | -6.2 (-18.1; 5.6) |
| Length of stay in hospital^b^, median days [IQR] | 6.5 [6; 9] | 14 [8;20] | -6 (-12; 0) |
| Length of stay in ICU^b^, median days [IQR] | 0 [0; 0] | 0 [0; 0] | 0 (0; 0) |
| Days on mechanical ventilator, median [IQR] | 0 [0; 0] | 0 [0; 0] | 0 (0; 0) |
| Days on HFNC^c^, median [IQR] | 3 [1; 7] | 4 [0;7] | 0 (-4; 3) |
| Adverse events, n (%) |  |  |  |
| Blood and lymphatic system disorders | 0 (0.0) | 0 (0.0) | 0.0 (0.0; 0.0) |
| Cardiac disorders | 1 (8.3) | 6 (37.5) | -29.5 (-57.6; -0.8) |
| Endocrine disorders | 1 (8.3) | 2 (12.5) | -4.2 (-26.7; 18.4) |
| Gastrointestinal disorders | 0 (0.0) | 0 (0.0) | 0.0 (0.0; 0.0) |
| Hepatobiliary disorders | 2 (16.7) | 1 (6.2) | 10.4 (-13.8; 34.6) |
| Infection | 0 (0.0) | 6 (37.5) | -37.5 (-61.2; -13.8) |
| Nervous system disorders | 1 (8.3) | 2 (12.5) | -4.2 (-26.7; 18.4) |
| Psychiatric disorders | 0 (0.0) | 1 (6.2) | -6.2 (-18.1; 5.6) |
| Respiratory disorders | 3 (25.0) | 6 (37.5) | -12.5 (-46.6; 21.6) |
| Skin and subcutaneous tissue disorders | 1 (8.3) | 2 (12.5) | -4.2 (-26.7; 18.4) |
| Vascular disorders | 0 (0.0) | 2 (12.5) | -12.5 (-28.7; 3.7) |

1. New episode of oxygen saturation ≤93% after the primary endpoint of a saturation >93% for at least 24 hours had been met.
2. Median days for the total study group.
3. High-flow nasal cannula.
