# Supplementary material for: Aerosolized Dornase Alfa (DNase I) for the Treatment of Severe Respiratory Failure in COVID-19: A Randomized Controlled Trial
Source: Open Forum Infect Dis. 2025 Apr 24;12(5):ofaf246. doi: 10.1093/ofid/ofaf246 (PMC12069806; doi:10.1093/ofid/ofaf246)
Supplement: ofaf246_Supplementary_Data [file ofaf246_supplementary_data.zip › Supplemental Table 2.docx]

Supplemental Table 2: The secondary outcomes for participants aged 64 years or younger.

| **Secondary outcome** | **Dornase alfa**  **(n=24)** | **Placebo**  **(n=24)** | **Difference in proportion**  **(95% CI) or Median (IQR)** |
| --- | --- | --- | --- |
| Deseased, n (%) | 0 (0.0) | 0 (0.0) | 0.0 (0.0; 0.0) |
| New episode of hypoxia^a^, n (%) | 4 (16.7) | 1 (4.2) | 12.5 (-4.4; 29.4) |
| Length of stay in hospital, median days [IQR] | 7 [5; 13] | 7.5 [5; 11] | -1 (-2; 3) |
| Length of stay in ICU^b^, median days [IQR] | 0 [0; 0] | 0 [0; 0] | 0 (0; 0) |
| Days on mechanical ventilator^b^, median [IQR] | 0 [0; 0] | 0 [0; 0] | 0 (0; 0) |
| Days on HFNC^c^, median [IQR] | 1.5 [0;6] | 2 [0;4] | 0 (-2; 3) |
| Adverse events, n (%) |  |  |  |
| Blood and lymphatic system disorders | 1 (4.2) | 0 (0.0) | 4.2 (-3.8; 12.2) |
| Cardiac disorders | 3 (12.5) | 3 (12.5) | 0.0 (-18.7; 18.7) |
| Endocrine disorders | 1 (4.2) | 1 (4.2) | 0.0 (-11.3; 11.3) |
| Gastrointestinal disorders | 5 (20.8) | 3 (12.5) | 8.3 (-12.6; 29.3) |
| Hepatobiliary disorders | 3 (12.5) | 3 (12.5) | 0.0 (-18.7; 18.7) |
| Infection | 7 (29.2) | 2 (8.3) | 20.8 (-0.4; 42.1) |
| Nervous system disorders | 4 (16.7) | 3 (12.5) | 4.2 (-15.8; 24.1) |
| Psychiatric disorders | 2 (8.3) | 1 (4.2) | 4.2 (-9.5; 17.8) |
| Respiratory disorders | 7 (29.2) | 5 (20.8) | 8.3 (-16.1; 32.7) |
| Skin and subcutaneous tissue disorders | 0 (0.0) | 3 (12.5) | -12.5 (-25.7; 0.7) |
| Vascular disorders | 1 (4.2) | 1 (4.2) | 0.0 (-11.3; 11.3) |

1. New episode of oxygen saturation ≤93% after the primary endpoint of a saturation >93% for at least 24 hours had been met.
2. Median days for the total study group.
3. High-flow nasal cannula.
