# Supplementary figures and images for: Aerosolized Dornase Alfa (DNase I) for the Treatment of Severe Respiratory Failure in COVID-19: A Randomized Controlled Trial
Source: Open Forum Infect Dis. 2025 Apr 24;12(5):ofaf246. doi: 10.1093/ofid/ofaf246 (PMC12069806; doi:10.1093/ofid/ofaf246)

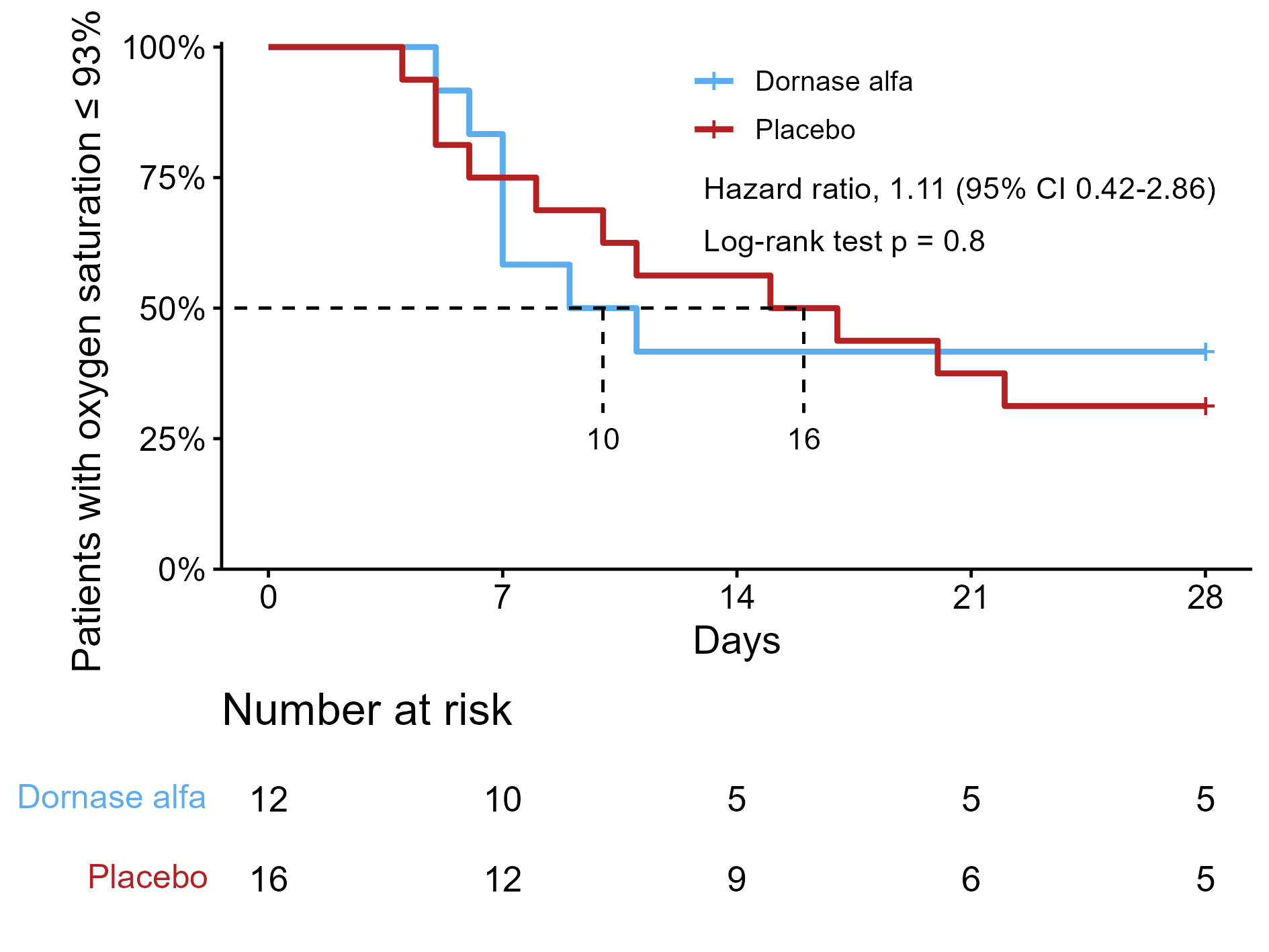

Supplement: ofaf246_Supplementary_Data [file ofaf246_supplementary_data.zip › Suppl Figure 1 A.tiff]

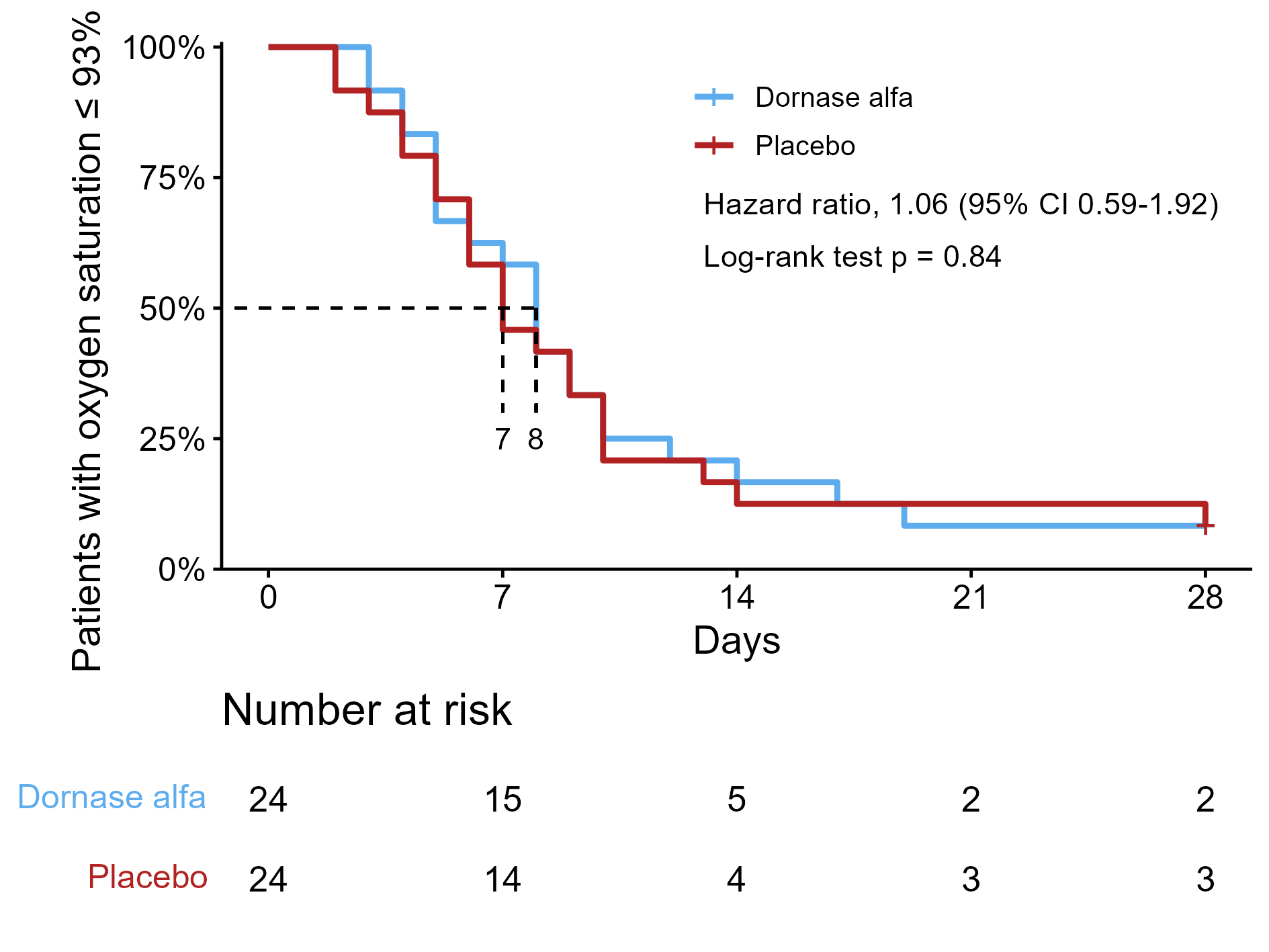

Supplement: ofaf246_Supplementary_Data [file ofaf246_supplementary_data.zip › Suppl Figure 1 B.tiff]
